# Molecular characterisation of the NSP4 gene of group A human rotavirus G2P[4] strains circulating in São Paulo, Brazil, from 1994 and 2006 to 2010

**DOI:** 10.1590/0074-02760150199

**Published:** 2015-09

**Authors:** Jéssica Wildgrube Bertol, Maria Clara Duarte Fregolente, Thabata Alessandra Ramos Caruzo, Márcio José da Silva, Veridiana Munford, Marco Aurélio Palazzi Sáfadi, Maria Lucia Rácz, Maria Silvia Viccari Gatti

**Affiliations:** 1 Universidade Estadual de Campinas, Instituto de Biologia, Departamento de Genética, Evolução e Bioagentes, Campinas, SP, Brasil; 2 Universidade da Cidade de São Paulo, São Paulo, SP, Brasil; 3 Universidade Estadual de Campinas, Centro de Biologia Molecular e Engenharia Genética, Campinas, SP, Brasil; 4 Universidade de São Paulo, Instituto de Ciências Biomédicas, Departamento de Microbiologia, São Paulo, SP, Brasil; 5 Faculdade de Ciências Médicas da Santa Casa de São Paulo, São Paulo, SP, Brasil

**Keywords:** human rotavirus, NSP4 genotypes, phylogenetic analysis, VP4/VP7/NSP4 linkage, Brazil

## Abstract

Group A human rotaviruses (HuRVA) are causative agents of acute gastroenteritis. Six viral structural proteins (VPs) and six nonstructural proteins (NSPs) are produced in RV-infected cells. NSP4 is a diarrhoea-inducing viral enterotoxin and NSP4 gene analysis revealed at least 15 (E1-E15) genotypes. This study analysed the NSP4 genetic diversity of HuRVA G2P[4] strains collected in the state of São Paulo (SP) from 1994 and 2006-2010 using reverse transcription-polymerase chain reaction, sequencing and phylogenetic analysis. Forty (97.6%) G2P[4] strains displayed genotype E2; one strain (2.4%) displayed genotype E1. These results are consistent with the proposed linkage between VP4/VP7 (G2P[4]) and the NSP4 (E2) genotype of HuRVA. NSP4 phylogenetic analysis showed distinct clusters, with grouping of most strains by their genotype and collection year, and most strains from SP were clustered together with strains from other Brazilian states. A deduced amino acid sequence alignment for E2 showed many variations in the C-terminal region, including the VP4-binding domain. Considering the ability of NSP4 to generate host immunity, monitoring NSP4 variations, along with those in the VP4 or VP7 protein, is important for evaluating the circulation and pathogenesis of RV. Finally, the presence of one G2P[4]E1 strain reinforces the idea that new genotype combinations emerge through reassortment and independent segregation.

Rotaviruses (RVs) are the most important etiological agents of severe acute gastroenteritis in children younger than five years of age (WHO 2013) and they cause an estimated 1.7 billion episodes of acute diarrhoea, leading to nearly 700,000 deaths worldwide annually (Liu et al. 2012, Walker et al. 2013). In Latin America, RV has been estimated to cause 6,302 deaths and 229,656 hospitalisations annually in 14 countries, including Brazil, in the absence of vaccination (Desai et al. 2012).

RVs belong to the Reoviridae family, Sedoreovirinae subfamily and genus *Rotavirus*(ICTV 2014). The nonenveloped virion is 100 nm in diameter and a triple-layered capsid surrounds the genome, which is composed of 11 segments of double-stranded RNA (dsRNA). These segments encode six viral structural proteins (VP1-4 and VP6-7) and six nonstructural proteins (NSP1-6) (Estes & Kapikian 2007, Greenberg & Estes 2009).

RVs are classified into eight species (A-H). Most strains that infect humans belong to group A (RVA); however, some group B, C and H strains have been associated with infections in humans (Matthijnssens & Van Ranst 2012, Matthijnssens et al. 2012). Based on the results of reverse transcription-polymerase chain reaction (RT-PCR) and sequence analyses, RVA strains are classified into the G and P genotypes, which represent the two type-specific outer capsid proteins, VP7 (glycoprotein) and VP4 (protease-sensitive protein), respectively. At least 27 G-types and 37 P-types have been described to date (Matthijnssens et al. 2011,Trojnar et al. 2013).

The most common G and P genotype combinations in human RVA (HuRVA) strains are G1P[8], G2P[4], G3P[8], G4P[8] and G9P[8] (Santos & Hoshino 2005, Greenberg & Estes 2009,CDC 2011). Although the prevalence of G2P[4] in Phase III trials with Rotarix^®^ in Brazil was reportedly low, some studies have shown the re-emergence of this genotype from 2005-2008 (Gurgel et al. 2007, Leite et al. 2008, Munford et al. 2009, Gómez et al. 2011). Additionally, the rate of G2P[4] detection has been decreasing since 2009 (Carvalho-Costa et al. 2011, Oliveira et al. 2012, Soares et al. 2012). This genotype fluctuation may be due to a natural phenomenon of re-emergence that occurs approximately every 10 years or may be related to the introduction of the Rotarix^®^ vaccine or both (Gómez et al. 2011).

The nonstructural RV protein NSP4, encoded by RV segment 10, was the first viral enterotoxin to be described (Ball et al. 1996). NSP4 is a multifunctional glycoprotein involved in viral morphogenesis and pathogenesis (Ball et al. 2005) that is important for the assembly of transiently enveloped double-layered particles (DLP) during RV replication (Taylor et al. 1996). NSP4 also promotes an increase in the intracellular calcium concentration, which results in chloride secretion into the intestinal lumen (Ball et al. 1996), the disruption of tight junctions (Tian et al. 1996) and, consequently, aqueous diarrhoea (Ball et al. 1996).

The gene that encodes NSP4 has been characterised into six (A-F) (Horie et al. 1997, Ciarlet et al. 2000, Ito et al. 2001, Mori et al. 2001) or 15 genotypes (E1-E15) (Papp et al. 2012). The previously described A, B and C genotypes are typically found in human RVs and correspond to the new E2, E1 and E3 genotypes, respectively (Matthijnssens et al. 2008a). As the diversity among NSP4 genotypes may be important for amino acid (aa) changes that could alter the virulence of HuRVA strains (Rajasekaran et al. 2008), it remains unclear whether NSP4 should be included in RV vaccination strategies because this protein also appears to play roles in immunity and protection (Ball et al. 2005, Araújo et al. 2007a).

Many studies have focused on molecular characterisation of the NSP4 gene of HuRVA collected from different regions of Brazil (Mascarenhas et al. 2006, Araújo et al. 2007b, Tavares et al. 2008, Nozawa et al. 2010, Fumian et al. 2011, Gómez et al. 2011); however, no studies have examined HuRVA strains collected in the state of São Paulo (SP). The aim of this study was to characterise the E genotypes and NSP4 aa sequences of 41 HuRVA strains from SP that were previously identified as G2P[4]. By using strains collected during distinct temporal periods, possible linkages between the VP7, VP4 and NSP4 genes were explored.

## SUBJECTS, MATERIALS AND METHODS

*Stool samples - *In total, 681 diarrheic stool samples were collected from children and adults hospitalised in SP in 1994 and from 2006-2010 (excluding 2008). Faecal suspensions (20% final volume) in Tris/calcium buffer (0.1 M Tris/HCl at pH 7.4 and 1.5 mM CaCl_2_) were centrifuged and the supernatants were immediately used or stored at -20ºC. RVs from the faecal samples were identified via enzyme immunoassay (EIA) (Munford et al. 2009).

*RNA extraction and genome segment amplification - *dsRNA was extracted using TRIzol reagent according to the manufacturer’s instructions (Invitrogen, USA) and stored at -20ºC until use.

The samples collected in 1994, 2006 and 2007 were previously characterised as G2P[4] using semi-nested multiplex RT-PCR (Munford et al. 2007, 2009, Sáfadi et al. 2010). The stool samples from 2009 and 2010 were characterised as G2P[4] using semi-nested RT-PCR with a One-Step RT-PCR kit (Qiagen) (Matthijnssens et al. 2008a).

The primers used for the amplification of segment 10 were GEN_NSP4Fb (5’-AAAGTTCTGTTCCGAGAGAGCG-3’) and GEN_NSP4Rb (5’-GACCRTTCCTTCCATTAACGTCC-3’), as described previously (Matthijnssens et al. 2008a). The 731-bp fragment was visualised under ultraviolet light after 1.5% agarose gel electrophoresis.

*Nucleotide sequencing - *The gene-specific PCR amplicons were subjected to genomic characterisation of E-types (NSP4) using a BigDye Terminator 3.1 kit and the same primers were employed in RT-PCR using an automated DNA capillary sequencer (ABI-PRISM 3700 DNA Analyzer, AB Applied Biosystems, USA; ABI PRISM DNA Sequencing Analysis Software; BigDye Terminator v.3.1 Cycle Sequencing Kit, Part No 4336919, AB Applied Biosystems).

*Sequence assembly and phylogenetic analysis - *The chromatogram sequencing files were analysed and aa alignments were constructed using BioEdit v.7.1.9 (Hall 1999). All of the consensus sequences were submitted for genotyping analysis using the web-based tool RotaC (Maes et al. 2009) (rotac.regtools.be). These sequences are available in the GenBank database under the accessions KC822323-KC822362.

Phylogenetic analyses were performed using MEGA v.6 (Tamura et al. 2011) and the genetic distances were calculated using the Kimura-2 correction parameter. A dendrogram was constructed using the neighbor-joining method (Matthijnssens et al. 2008a) with 1,000 bootstrap repetitions.

The E1 and E2 genotype patterns included in the phylogenetic tree were selected according to the higher percentages of similarity with sequences obtained from RotaC.

## RESULTS

Of 113 RV samples that tested positive, as determined by EIA, 80 (71%) were successfully genotyped for G and P by RT-PCR. A high predominance of the G2P[4] combination (78 samples, 97.5%) was observed in these 80 samples. The other two samples were characterised as G3P[6] and G2P[8]. Among the 78 G2P[4] samples, 41 (52.6%) were sequenced for the NSP4 gene. The results indicated that 97.6% of these 41 strains were of the E2 genotype and that only one (2.4%) was of the E1 genotype.

**Fig. 1:**
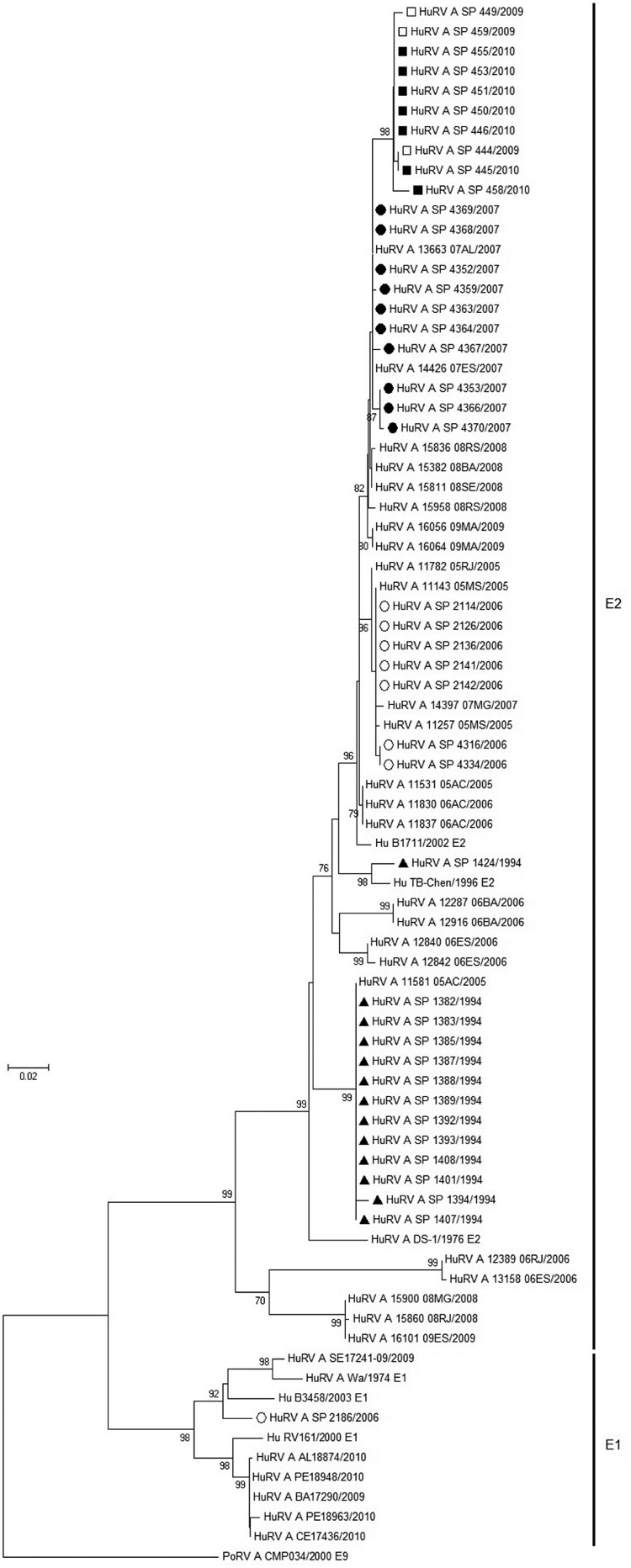
: phylogenetic analysis of nucleotide sequences of the nonstructural protein 4 of G2P[4] human rotavirus group A (HuRVA) strains circulating in the state of São Paulo, Brazil. The porcine RV E9 strain CMP034 (DQ534017) was used as the outgroup. A neighbor-joining tree with a 1,000-fold bootstrap was constructed with the evolutionary distances computed using the Kimura 2-parameter method. The bootstrap values above 70% are indicated. The bars are in units of substitution per nucleotide. The symbols indicate the sample collection year of the strains from this study. ▲: 1994 strains; ○: 2006 strains; ●: 2007 strains; □: 2009 strains; ■: 2010 strains; Po: porcine.

Different HuRVA strains detected in Brazilian states were used to construct a phylogenetic tree along with RV reference strains of the E genotypes (Matthijnssens et al. 2011) and strains with major similarities, as assessed by RotaC. The phylogenetic tree produced distinct clusters, grouping most strains by their genotype and collection year (Fig. 1). As expected, E1 strain SP 2186 clustered with E1 strains from different Brazilian states; however, this strain did show 96.5% nucleotide similarity with the B3458 (EF990712.1) strain from Belgium.

The same cluster pattern occurred with the E2 strains, with the year of sample collection influencing the phylogenetic tree structure. The strains from 1994 formed a branch with the 11581 05AC strain from 2005, although strain SP1424, which was also collected in 1994, was grouped in a different branch with only strain TB-Chen from 1996. The strains collected from 2006-2010 clustered with the HuRV-A/B1711 strain (collected in 2002), a G6P[6]E2 strain with a DS-1-like lineage.

Most of the strains from SP clustered together with strains from other Brazilian states. Only one branch was solely composed of strains obtained in this study (during the years 2009 and 2010).

**Fig. 2:**
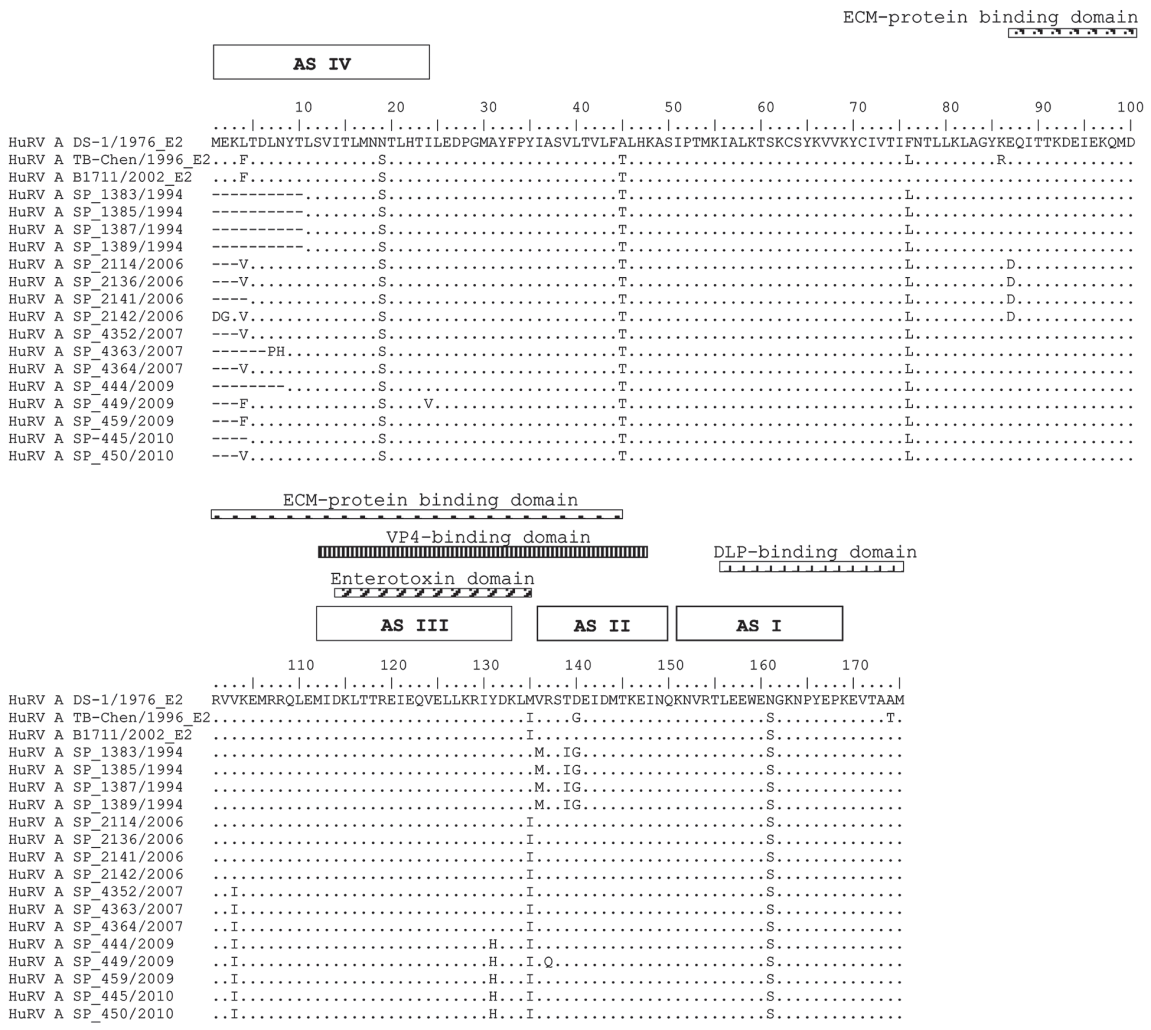
: comparison of the deduced amino acid (aa) sequences of the nonstructural protein 4 of G2P[4]E2 human rotavirus group A (HuRVA) strains detected in this study. The sequences of DS-1, TB-Chen and B1711 with the GenBank accessions AF174305, AY787650 and EF554091, respectively, were used as the reference strains. The dots indicate the identity of the DS-1 strain and the aas that differ from DS-1 are shown for the remaining strains using the single-letter aa code. The extracellular matrix (ECM)-protein binding domain (87-145), viral structural proteins (VP)4-binding domain (112-148), enterotoxin domain (114-135), double-layered particles (DLP)-binding domain (156-175), antigenic site (AS) IV (1-24), AS III (112-133), AS II (136-150) and AS I (152-169) are indicated by overlines.

Among the 40 deduced aa sequences of the HuRVA E2 NSP4 gene, 16 were aligned to represent the primary observed aa variations (Fig. 2). The alignment results revealed the prevalence of variations at antigenic sites II and III, the VP4-binding domain, the enterotoxin domain and the extracellular matrix-protein binding domain.

Some nucleotide polymorphisms led to aa changes in all of the strains collected in a given year (Table). Only the strains collected in 2006 encoded a different aa at position 87 (E→D). The same type of change was observed at positions 136 (V→M) and 139 (T→I) for strains from 1994. Positions 103 (V→I) and 131 (Y→H) exhibited aa changes in 2007 and 2009, respectively, and these changes were maintained in the strains isolated in later years. Finally, with regard to position 135, the strains from 1994 showed the same aa as the RV reference strain DS-1 (M), whereas the strains from the other years showed the same aa as RV patterns TB-Chen and B1711 (I).

**TABLE:** The amino acid*a* (aa) differences of nonstructural protein 4 (E2) from human rotavirus group A circulating in the state of São Paulo, Brazil, according to the sample collection year (1994 and 2006 to 2010)

aa	DS-1	TB-Chen	B1711	1994	2006	2007	2009	2010
19	N	S	S	S	S	S	S	S
45	A	T	T	T	T	T	T	T
76	F	L	F	L	L	L	L	L
87	E	E	E	E	D	E	E	E
103	V	V	V	V	V	I	I	I
131	Y	Y	Y	Y	Y	Y	H	H
135	M	I	I	M	I	I	I	I
136	V	V	V	M	V	V	V	V
139	T	T	T	I	T	T	T	T
140	D	G	D	G	D	D	D	D
161	N	S	S	S	S	S	S	S

*a*: the numbering is based on DS-1 (GenBank accession AF174305).

## DISCUSSION

This study analysed 41 HuRVA strains classified as G2P[4]; the NSP4 sequences were genotyped as E1 or E2, allowing for an investigation of the linkage between VP4, VP7 and NSP4. The deduced aa sequences were determined and variations were observed at different sites of the NSP4 coding sequence.

The results suggested a linkage between VP7, VP4 and NSP4, as 40 (97.6%) G2P[4] HuRVA strains displayed the E2 genotype. In contrast, only one strain (2.4%) displayed the E1 genotype.

A new full genome-based classification system has established 15 NSP4 genotypes, as determined by nucleotide sequencing and phylogenetic analysis. This system establishes NSP4 genotype B (Wa-like) as the E1 genotype and genotype A (DS-1-like) as the E2 genotype, which is the most diverse; the E3 genotype strains include the previous NSP4 genotype C (AU-1-like) (Matthijnssens et al. 2008a, b). Intragenotypic diversity has also been reported for the majority of RV strains that infect other species (Papp et al. 2012).

Most HuRVA strains are NSP4 genotype E2 or E1. Genotype E2 appears to be closely related to G2P[4] strains, whereas genotype E1 appears to be most closely related to G1P[8] strains, more distantly related to G3P[8], G4P[8] and G9P[8]/P[6] (Araújo et al. 2007a, Tatte et al. 2010, Fredj et al. 2014) and rarely related to G2P[4] strains (Benati et al. 2010). These results are consistent with those of studies suggesting a linkage among the VP7, VP4 and NSP4 genes. Nonetheless, RV genes may also segregate independently in nature (Maunula & Von Bonsdorff 2002).

NSP4 phylogenetic analysis revealed clustering of the sequences according to genotype and sample collection year and the reference strains for each genotype were also clustered. The phylogenetic tree showed E2 intragenotype variation due to the presence of different branches formed by the strains from the same collection year. The same pattern of intragenotypic variation has been observed in E1 strains in Mexico and the authors attributed this finding to the accumulation of single point mutations in the NSP4 gene (González-Ochoa et al. 2013).

The HuRVA strains from different Brazilian states also clustered with the strains from this study. However, strains 16101 09ES/2009, 15900 08MG/2008 and 15860 08RJ/2008 formed a different branch in the phylogenetic tree. Gómez et al. (2011) has reported that these three Southeast Brazilian strains cluster into a monophyletic group due to their common region of origin. Therefore, this group would be expected to cluster together with the Southeast Region strains examined in the present study. This finding reinforces the intragenotypic variation exhibited by the NSP4 gene. Finally, the results did not provide evidence of the segregation of strains according to different Brazilian regions and this mixed distribution emphasises that circulation of the HuRVA genotypes in Brazil is homogeneous, despite the wide expanse of this country’s territory.

The G2P[4]E2 strains from this study segregated into groups according to the collection year, a finding that has also been observed by Gómez et al. (2011), who analysed G2P[4]E2 strains from different Brazilian states. However, when the strains in the present study were analysed together with the strains from Gómez et al. (2011), the resulting tree formed mixed branches, regardless of the collection year. This intragenotypic variation according to the sample collection year leads to changes in aa sequence that could also be related to the collection year, thus supporting the cluster formation observed in the phylogenetic tree. In addition, these temporal variations can occur due to point mutations in the NSP4 gene.

Most of the variations in NSP4 aas from E2 strains occurred in the carboxy-terminal region, including the VP4-binding domain, and the same result has been observed in studies comparing NSP4 aas from E1 strains (González-Ochoa et al. 2013, Fredj et al. 2014). This finding suggests that NSP4 is involved in viral morphogenesis and that additional studies are necessary to understand how these variations in the NSP4 protein may interfere with the structural conformation of the interaction sites between NSP4 and VP4.

Most mutations were observed in the interspecies variable domain (ISVD) (aa 135-141). Within the ISVD domain, the presence of a glycine at position 140 defines the E2 subgenotype 1. Therefore, all of the strains from 1994 belong to subgenotype 1 and the others are classified as subgenotype 2 (Deepa et al. 2007, Fredj et al. 2014). Additionally, the residues D143, M144 and K146 and the last 16 aas, including the carboxy-terminal methionine, in the VP4- and DLP-binding domains, respectively, are highly conserved among all of the strains (Fredj et al. 2014). Previous studies have demonstrated that this conserved carboxy-terminal region (16-20 aa) is important for DLP-binding activity (O’Brien et al. 2000). With regard to the specific M-I mutation at position 135 in the strains from this study, the mutation most likely occurred between 1994 and 1996 because the DS-1 and TB-Chen strains were collected in 1976 and 1996, respectively.

Although NSP4 is a highly conserved protein, point mutations and genetic drift have led to aa changes over time. Because NSP4 induces a humoral immune response (Johansen et al. 1999), these changes may allow RVs to adapt or to escape host immunity. Therefore, variations in the NSP4 protein, together with variations in the VP4 and VP7 proteins, could be used to monitor RV circulation.

Finally, based on the prevalence of E2 genotypes in G2P[4] HuRVA strains, possible linkage among the G2, P[4] and E2 genotypes was confirmed. The detection of one E1 G2P[4] strain reinforces the idea that new genotype combinations emerge through reassortment and independent segregation.

In conclusion, we have demonstrated NSP4 genetic diversity in HuRVA G2P[4] strains collected in SP over the course of 12 years. The results reinforce the observed NSP4 intragenotype variation, which can likely be attributed to single point mutations and genetic drift events. The predominance of NSP4 aa changes in the VP4-binding domain emphasise the role of NSP4 in viral morphogenesis. Genomic and aa monitoring of circulating RVA strains may aid in identifying the occurrence of variations that might influence host-pathogen relationships. Moreover, the monitoring of genetic diversity among RVA strains is important for predicting the possible reemergence of strains after the introduction of vaccines against RVA.
